# Dissociated Mechanisms of Extracting Perceptual Information into Visual Working Memory

**DOI:** 10.1371/journal.pone.0014273

**Published:** 2010-12-10

**Authors:** Zaifeng Gao, Jie Li, Jun Yin, Mowei Shen

**Affiliations:** Department of Psychology and Behavioral Sciences, Zhejiang University, Hangzhou, China; University of Minnesota, United States of America

## Abstract

**Background:**

The processing mechanisms of visual working memory (VWM) have been extensively explored in the recent decade. However, how the perceptual information is extracted into VWM remains largely unclear. The current study investigated this issue by testing whether the perceptual information was extracted into VWM via an integrated-object manner so that all the irrelevant information would be extracted (object hypothesis), or via a feature-based manner so that only the target-relevant information would be extracted (feature hypothesis), or via an analogous processing manner as that in visual perception (analogy hypothesis).

**Methodology/Principal Findings:**

High-discriminable information which is processed at the parallel stage of visual perception and fine-grained information which is processed via focal attention were selected as the representatives of perceptual information. The analogy hypothesis predicted that whereas high-discriminable information is extracted into VWM automatically, fine-grained information will be extracted only if it is task-relevant. By manipulating the information type of the irrelevant dimension in a change-detection task, we found that the performance was affected and the ERP component N270 was enhanced if a change between the probe and the memorized stimulus consisted of irrelevant high-discriminable information, but not if it consisted of irrelevant fine-grained information.

**Conclusions/Significance:**

We conclude that dissociated extraction mechanisms exist in VWM for information resolved via dissociated processes in visual perception (at least for the information tested in the current study), supporting the analogy hypothesis.

## Introduction

Visual working memory (VWM) maintains and manipulates a limited set of visual objects which are transferred from visual perception [1], [2]. As one of the most important information processing modules of human beings, it plays a critical role in many high-level cognitive activities, such as visual search, motion, and transsaccadic perception [3]–[6]. So far, much research has been conducted to uncover its underlying mechanisms, such as VWM capacity, storage unit, and neural substrates for maintaining visual objects [1], [7]–[10], and information consolidation, retrieval and comparison in VWM [11]–[13]. However, how the information stored in VWM is extracted from the perceptual objects remains largely unknown. Here we addressed this issue focusing on how high-discriminable information and fine-grained information are extracted into VWM by manipulating the type of task-irrelevant information in a change detection task.

Previous VWM research showed that both bottom-up and top-down factors have an influence on the extraction of object information into VWM. Visual transit such as abrupt onset of object [14] and bottom-up Gestalt perceptual organization [15], results in a higher probability for the objects to be selected and transferred into VWM. Similarly, a cue, whether be a pre-cue, a simultaneous cue, or a post-cue relative to the onset of memory array, guides visual attention to the location of the object, and the corresponding information is considerably better selected and retained in VWM [16]–[18]. In addition, fMRI research reveals that the processes of feature selection in a visual search task share common neural substrates with VWM extraction and consolidation [19], [20]. These studies greatly help us understand the way visual attention extracts perceptual information into VWM; however, they do not answer what exactly happens when the perceptual information begins to be extracted into VWM. This is an important question not only because we should first extract information before other further VWM operations in everyday life, but also because it will greatly enable us to better understand how visual perception and VWM interact, which is a far from clear issue.

To this end, the current research investigated the VWM extraction process by testing three possible hypotheses. First, VWM extraction may follow an object-based selection manner (*object hypothesis*). Specifically, once a feature dimension of an object is selected, the other dimensions will also be automatically extracted. This hypothesis is implicitly assumed by most of the early VWM research [1], [21]–[24], since it fits beautifully with studies on object-based attention [25]–[27] and the ‘object file’ theory in general [28]–[30]. Indeed, this hypothesis also gets direct support from behavioral [1], [12], [22], [31], [32], ERP [33]–[35] and fMRI [36] studies, by demonstrating that the irrelevant feature dimension (e.g., shape, orientation) is also encoded into VWM even only one target dimension of the objects needs to be maintained. Contrast to the object hypothesis, however, two recent studies implied that VWM may only select the target dimension of the objects without selecting the irrelevant dimensions (*feature hypothesis*). By exploring the consolidation and the maintenance phases of VWM for objects formed by two basic feature dimensions, researchers found that only the target dimension was extracted and stored yet no storage trace for the irrelevant one [37], [38].

Finally, a similar information extraction mechanism may be shared between visual perception, which selects features from the outside scene, and VWM, which extracts information from the perceptual object (*analogy hypothesis*). Recent neuroimaging studies provided evidence for this possibility. In addition to the facts that largely overlapping neural sources are revealed between the perceptual selection and VWM extraction [19], [20], recent studies also consistently showed that VWM maintenance is supported by the same cortical regions that encode the sensory information to be remembered [37], [39]–[43], although VWM is generally considered as a higher level process maintaining the final outputs from visual perception. Therefore, a similar information extraction mechanism may be intuitively recruited between visual perception and VWM.

The information selection process in visual perception has traditionally been dichotomized into two stages [44]–[48], albeit there are still controversies (see [47], [50]–[52] for example). At the first stage, the distinct basic features (e.g., high-discriminable color, shape) are preattentively processed via an automatic and parallel processing, following the bottom-up visual pathway with coarse, pre-categorical information as the output. At the second stage, the fine-grained information (e.g., conjunction between features; a small gap on the frame of a circular ring; for a review, see [49]) is serially encompassed into perception proceeding in a top-down fashion via focal attention. Thereby, the analogy hypothesis of VWM extraction predicts that the high-discriminable information should be automatically selected into VWM regardless of task demand, which is similar to the prediction of the object hypothesis and thus receives support from studies which meet the object hypothesis prediction since the stimuli used in these studies were formed by basic features. Furthermore, it predicts that the extraction of fine-grained information requires top-down processing, which is similar to the prediction of the feature hypothesis but has not been examined before.

It is critical to note that the above three hypotheses have distinct predictions on the extraction of the irrelevant dimension. In particular, the object hypothesis predicts that the irrelevant information will always be extracted regardless of the information type; the feature hypothesis claims that it can not be extracted at all; whereas the analogy hypothesis suggests the extraction of the irrelevant dimension should depend on the type of information processed at the perceptual stage. Therefore, our goal of the current study was to investigate the VWM extraction mechanism by examining whether a specific type of the irrelevant information can be extracted into VWM automatically regardless of the task demand. Two types of visual information mentioned above (i.e., high-discriminable information and fine-grained information) were chosen to represent the information processed at the two stages of visual perception.

To get converging evidence on the extraction mechanism, especially extraction evidence about the fine-grained information which may not be extracted automatically, except for analyzing the behavioral performance we also recorded event-related potentials (ERP). Specifically, we focused on an ERP component N270 (or N2), which reflects the mismatch between the representation in VWM and the incoming perceptual input [33], [35], [53], [54], as a neural index of interest in the current study. Previous research showed that when a probe item is different from the memorized item in a one-item change detection task, a negative component will be elicited at around 270 ms (N270) after the probe item's onset, predominantly exhibiting a frontal distribution [33], [55], [56] with the source at anterior cingulate cortex (ACC) [57]. Furthermore, it is found that N270 is not elicited when the blank interval between the two items is 150 ms, but is elicited at longer intervals (e.g., 500 ms) [58] as well as in a classical Sternberg probe-matching working memory task [54], suggesting N270 has a functional bond with VWM. Here we asked the participants to remember the target dimension while ignoring the irrelevant one in the experiment. If the irrelevant information is automatically extracted into VWM, the mismatch between the memorized item and the probe induced by the change of the irrelevant dimension will influence the behavioral performance and elicit N270; otherwise, there will be no impact on the performance or no N270 elicitation. To preview the results, we found that only the irrelevant change of high-discriminable information affected the performance and elicited N270, supporting the analogy hypothesis.

## Experiment 1a: Automatic Extraction of Irrelevant High-discriminable Information

Previous N270 research showed that basic shapes as the irrelevant dimension can be automatically extracted into VWM [34], [35], [59], which supports both the object hypothesis and the analogy hypothesis. However, behavioral and fMRI results by using distinctive colors as the irrelevant dimension are quite divergent (see [1], [12], [22] for automatic extraction; but see [37], [38] for non-automatic extraction). Therefore, to start with, we tested whether a distinctive color can be automatically extracted by VWM even when it is irrelevant to the task.

### Methods

#### Participants

Participants were 17 (10 females) right-handed students (Mean age 24.3 years) from Zhejiang University. They reported no history of neurological problems, with normal or corrected-to-normal vision. All participants provided written and informed consent before experiments, and all procedures were approved by the Research Ethics Board of Zhejiang University.

#### Stimuli

**Figure 1:**
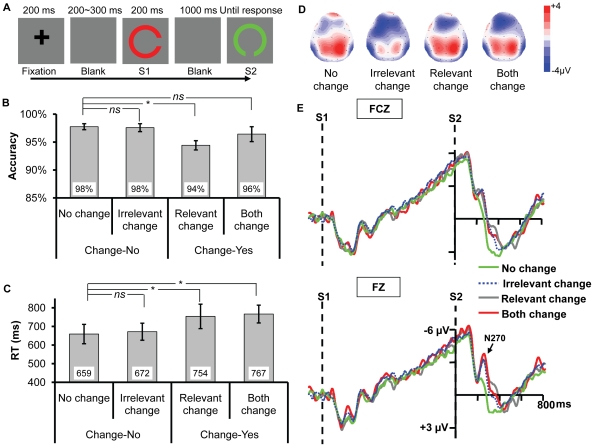
Example of an experimental trial and the results for Experiment 1a. (A) Illustration of the time course of a trial in Experiment 1a. Both the relevant (gap orientation) and irrelevant dimensions changed (color). (B) and (C) Accuracy and reaction times (RT) for the four types of change. The * and *ns* show the result of planned contrast between the three types of change and no change. * means the difference is significant, whereas *ns* means the difference is nonsignificant. (D) The frontal distribution of the ERP response at 249 ms (N270) following the onset of S2 for each condition. (E) The ERPs recorded at FZ and FCZ. All the three types of change elicited N270 relative to no change.

Landolt-Cs were adopted as stimuli (see Figure 1A, 1.01°×1.01° of visual angle for the circle, 0.25° of visual angle for the gap's height from a viewing distance of 70 cm). The orientation of a landolt-C was selected from a set of 8 orientations: 0°, 45°, 90°, 135°, 180°, 225°, 270° and 315°. The color of a landolt-C was selected from a set of 7 colors: red (255, 0, 0 in RGB value), green (0, 255, 0), blue (0, 0, 255), violet (255, 0, 255), yellow (255, 255, 0), black (0, 0, 0), and white (255, 255, 255). The stimuli were presented at the center of a 17 inch monitor with grey (128, 128, 128) background.

#### Procedure & Design

After a variable delay ranging from 1000 to 1400 ms, a fixation was displayed for 200 ms followed by 200∼300 ms blank interval. Then Stimulus 1 (S1) was displayed for 200 ms, followed by a 1000 ms blank period, and finally Stimulus 2 (S2) was presented until a response was initiated (see Figure 1A). Participants were instructed to remember the orientation of the gap in a landolt-C while ignoring the color, and justify whether the orientation of S2 was identical to S1. Seated in a chair in a sound- and electro-shielded room, the participants were instructed to press “F” on the keyboard for the target-change condition and “J for no-change condition. Both response accuracy and reaction times (RT) were emphasized and recorded.

Three mainly interested conditions were included: S2 was either the same as S1 (*no change*), or different from S1 in color (*irrelevant change*, the key condition we focused on), or different from S1 in orientation (*relevant change*). To control the probability of change and no-change on the target dimension, S1 and S2 different in both color and orientation (*both change*) as a fourth condition was also added in. There were 320 trials in total which were presented randomly, with 80 trials in each condition. The experiment was divided into 4 blocks with 8-minute break in between, and lasted about 45 minutes totally.

#### Electrophysiological Recording and Analysis

The EEG was recorded from 32 scalp sites using Ag/AgCl electrodes mounted in an elastic cap. All recordings were initially referenced to the left mastoid, and re-referenced offline to the average of the left and right mastoids. Vertical electrooculogram (VEOG) and horizontal electrooculogram (HEOG) were recorded with two pairs of electrodes, one pair placed above and below the left eye, and the other pair placed beside the two eyes. All interelectrode impedances were maintained below 5 KΩ. The EEG and EOG were amplified by SynAmps using a 0.05–100 Hz bandpass and continuously sampled at 1000 Hz/channel for off-line analysis. Electrooculogram artifacts were corrected using the regression method [60]. Additional artifact rejection was applied to epochs with EEG amplitude exceeding ±75 µV, which accounted for the exclusion of an average of 11.7% of trials. Two male participants were removed from further analysis for too many artifacts (over 30% of trials). The EEG and EOG were digitally filtered offline with a 0.05–30 Hz bandpass filter. The EEG was segmented into 2100-ms epochs starting from 100 ms before S1 onset, which was used as the baseline.

Based on previous studies [33]–[35], [61] and scrutiny of the present N270 distribution, the statistical analysis was restricted to the frontal regions (see Figure 1D). The mean amplitudes of N270 for the time window of 220–320 ms at sites FZ and FCZ were tested. Electrodes (FZ, FCZ) and change type (no change, irrelevant change, relevant change, and both change) were taken as factors for repeated two-way ANOVA. Greenhouse-Geisser correction was adopted where the degrees of freedom had more than one level (the uncorrected degrees of freedom were presented for simplicity). Planned contrast was conducted by comparing irrelevant change, relevant change, and both change with no change, to evaluate N270 and the influence of mismatch on accuracy and RT. For N270 and RT only the correct-response trials were tested.

### Results and Discussion

#### Behavioral Data

A one-way ANOVA by taking change type as a factor yielded significant main effects of change type on accuracy and RT (Figure 1B & Figure 1C), *F*(3,42) = 6.913, *p* = 0.002, *F*(3,42) = 15.920, *p*<0.001, respectively. Planned contrast showed that there was no difference between irrelevant change and no change on accuracy or RT, *p*s >0.2. The accuracy of relevant change was lower than that of no change, *F*(1,14) = 10.136, *p* = 0.007, suggesting that the change of gap orientation is harder to detect compared to no change. However, no difference was revealed on accuracy between both change and no change, *F*(1,14) = 4.350, *p*>0.05, which implied that when both relevant and irrelevant dimensions changed, the change of the gap orientation was easier to detect relative to when only the relevant dimension changed. Therefore, the change of the irrelevant dimension exerted an influence on the change detection of target dimension by a seemingly facilitation effect. Indeed, the accuracy of both change condition was considerably better than that of relevant change condition, *t*(14) = 2.256, *p*<0.05. The RTs of relevant change and both change were longer than that of no change, *F*(1,14) = 31.601, *p*<0.001, *F*(1,14) = 21.377, *p*<0.001, respectively.

#### ERP Data

As evident in Figure 1E, all the three types of change elicited a more negative component N270. The two-way ANOVA revealed a significant main effect of change type, *F*(3,42) = 4.467, *p* = 0.015, and a significant main effect of electrodes, *F*(1,14) = 6.617, *p* = 0.022; the interaction between the two factors was nonsignificant, *F*(3,42) = 1.953, *p* = 0.169. Planned contrast showed that the amplitude of irrelevant change was higher than that of no change, *F*(1,14) = 18.307, *p* = 0.001, suggesting that the irrelevant color change elicited N270. Meanwhile, both the amplitudes of relevant change and both change were higher than that of no change, *F*(1,14) = 10.949, *p* = 0.005, *F*(1,14) = 9.863, *p* = 0.007, respectively, indicating N270 was always elicited when the target dimension changed. No difference was found among irrelevant change, relevant change and both change, *F*(2,28) = 0.004, *p* = 0.995, indicating the task demand did not modulate the N270 amplitude.

The current experiment showed that the irrelevant change facilitated the change detection of gap orientation and elicited N270. It thus provided converging evidence suggesting that the irrelevant color is automatically extracted into VWM. Someone may argue that though the retention interval (1000 ms) is well matched with the parameter conventionally adopted in VWM research (e.g., [10], [22]), iconic memory still plays a role in Experiment 1a since the task is very simple, which thus confounds our findings. To rule out this alternative, we ran a control experiment by adding a backward mask after S1's offset to erase any iconic memory residue (which also stopped the consolidation process of S1 into VWM [11]).

## Experiment 1b: S1 with Backward Mask

### Methods

**Figure 2:**
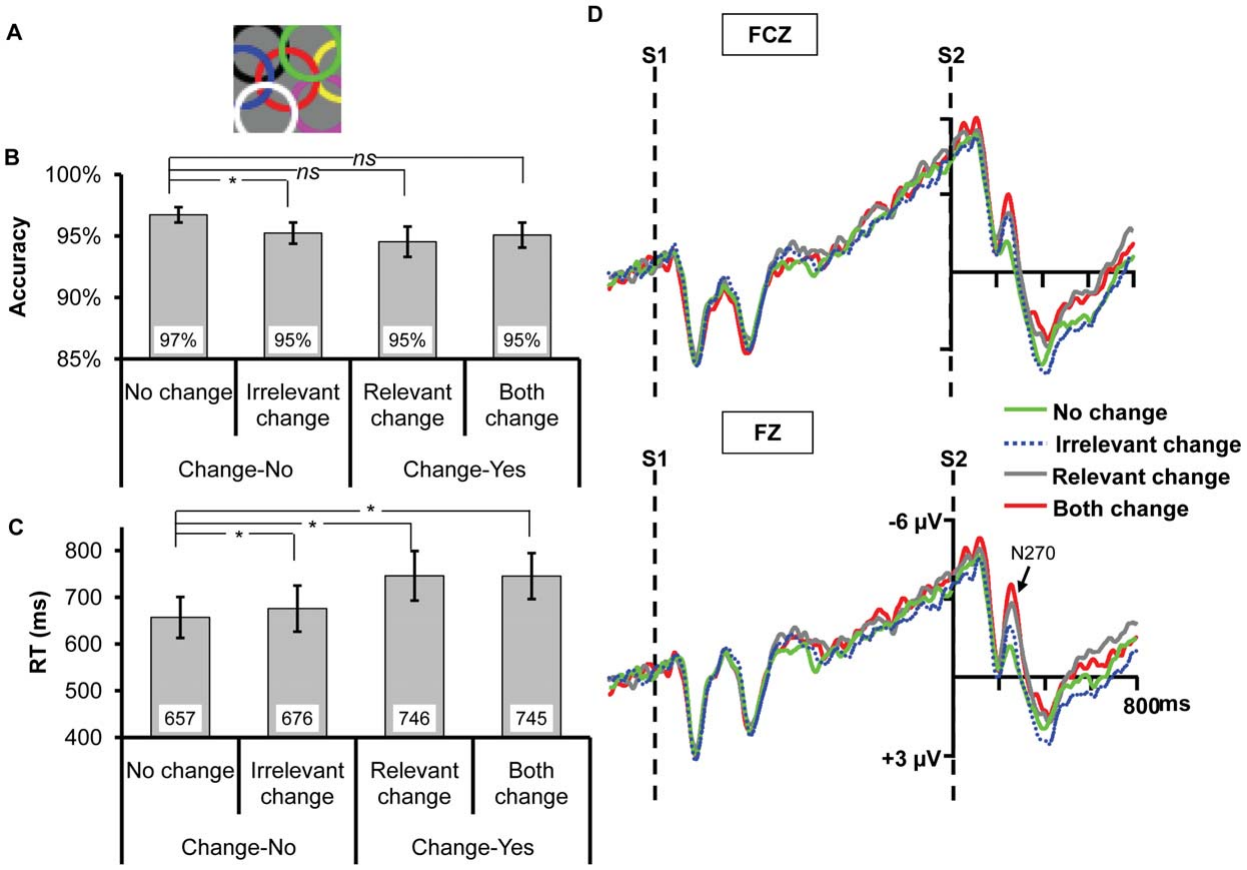
Example of the mask and the results for Experiment 1b. (A) Pattern mask used in the current experiment. (B) and (C) Accuracy and reaction times (RT) for the match-mismatch task, respectively. The * and *ns* show the result of planned contrast between the three types of change and no change. * means the difference is significant, whereas *ns* means the difference is nonsignificant. (D) The ERPs recorded at FZ and FCZ. All the three types of change elicited N270 relative to no change.

Sixteen students (5 females) participated in the experiment. A colored pattern mask (see Figure 2A) was displayed for 100 ms immediately after the offset of S1. All participants provided written and informed consent before experiments, and all procedures were approved by the Research Ethics Board of Zhejiang University. The mean amplitudes of N270 for the time window of 220–290 ms at sites FZ and FCZ were analyzed since it was more evident in this time window. The other aspects were identical to Experiment 1a.

### Results and Discussion

#### Behavioral Data

The overall accuracy in the current experiment (0.95) was slightly lower than that in Experiment 1a (0.97), suggesting that the mask blocked certain information albeit it was not significant (*t*(29) = 1.16, *p* = 0.256) which may due to between-experiment variance. A one-way ANOVA showed a significant main effect of change type on RT (Figure 2C), *F*(3,45) = 23.293, *p*<0.001; yet no effect on accuracy (Figure 2B), *F*(3,45) = 1.343, *p = *0.272. Planned contrast showed that the accuracy of irrelevant change was significantly lower than that of no change, *F*(1,15) = 4.672, *p = *0.047, and the RT of irrelevant change was also considerably longer than that of no change, *F*(1,15) = 4.770, *p* = 0.045, suggesting the change of irrelevant dimension impaired the performance. Though the accuracy under relevant change and both change seemed to be lower than no change, yet the differences between them were not significant, all *p*s >0.1. Both the RTs of relevant change and both change were slower than that of no change, *F*(1,15) = 21.165, *p*<0.001, *F*(1,15) = 27.944, *p*<0.001, respectively.

#### ERP Data

Replicating the result in Experiment 1a, Figure 2D showed that the irrelevant color change elicited N270. The two-way ANOVA revealed a significant main effect of change type, *F*(3,45) = 3.831, *p* = 0.034; the other ones were nonsignificant, all *p*s >0.25. Planned contrast showed that the amplitude of irrelevant change was higher than that of no change, *F*(1,15) = 5.154, *p* = 0.038, suggesting that the irrelevant color change elicited N270. Meanwhile, the amplitudes of relevant change and both change were higher than that of no change, *F*(1,15) = 7.667, *p* = 0.005, *F*(1,15) = 6.419, *p* = 0.023, respectively, indicating N270 was elicited once the target dimension changed. No difference was found among irrelevant change, relevant change and both change, *F*(2,32) = 0.905, *p* = 0.394.

By presenting a backward mask to erase any residual iconic memory trace of S1, again both behavioral and ERP results suggest that the irrelevant high-discriminable dimension is automatically selected into VWM. Therefore, Experiment 1 did not support the feature hypothesis. Besides, contrast to the facilitation effect on the performance caused by the irreverent dimension change in Experiment 1a, Experiment 1b showed an impairment effect. This may be because the representation of gap orientation was less robust than that in Experiment 1a for its consolidation process was stopped by the mask, which led the participants to judge as "change" even only the irrelevant dimension changed. However, in the following experiments which aimed at exploring the automatic extraction of the fine-grained information, we did not add a backward mask since we hoped to maximize the chance for the extraction of the fine-grained information considering the analogy hypothesis predicted that it would not be extracted automatically.

## Experiment 2a: Non-automatic Extraction of Irrelevant Fine-grained Information

In the current experiment, we explored whether the automatic selection in VWM was limited to the output of parallel perceptual processing (analogy hypothesis), or could be extended to the fine-grained information (object hypothesis). We hence simply switched the task relevant dimension and irrelevant dimension used in Experiment 1, and asked participants to remember the color of a landolt-C while ignoring its gap orientation. Previous research indicated that the processing of landolt-C's orientation requires focal attentive processing ([62], [63]; see [49] for a review), it is thus a type of detailed information.

### Methods

Fourteen (7 females) students (mean age 24.0 years old) participated in the experiment. All participants provided written and informed consent before experiments, and all procedures were approved by the Research Ethics Board of Zhejiang University. The participants were required to remember the color (relevant dimension) of S1, and judge whether the color of S2 was identical to that of S1 while ignoring the gap orientation (irrelevant dimension). Thereby, the irrelevant change meant that S2 differed from S1 in orientation only, while the relevant change meant that S2 differed from S1 in color only. The other aspects of Experiment 2a were identical to Experiment 1a. An average of 11.7% of trials was excluded from further analysis.

### Results and Discussion

#### Behavioral Data

**Figure 3:**
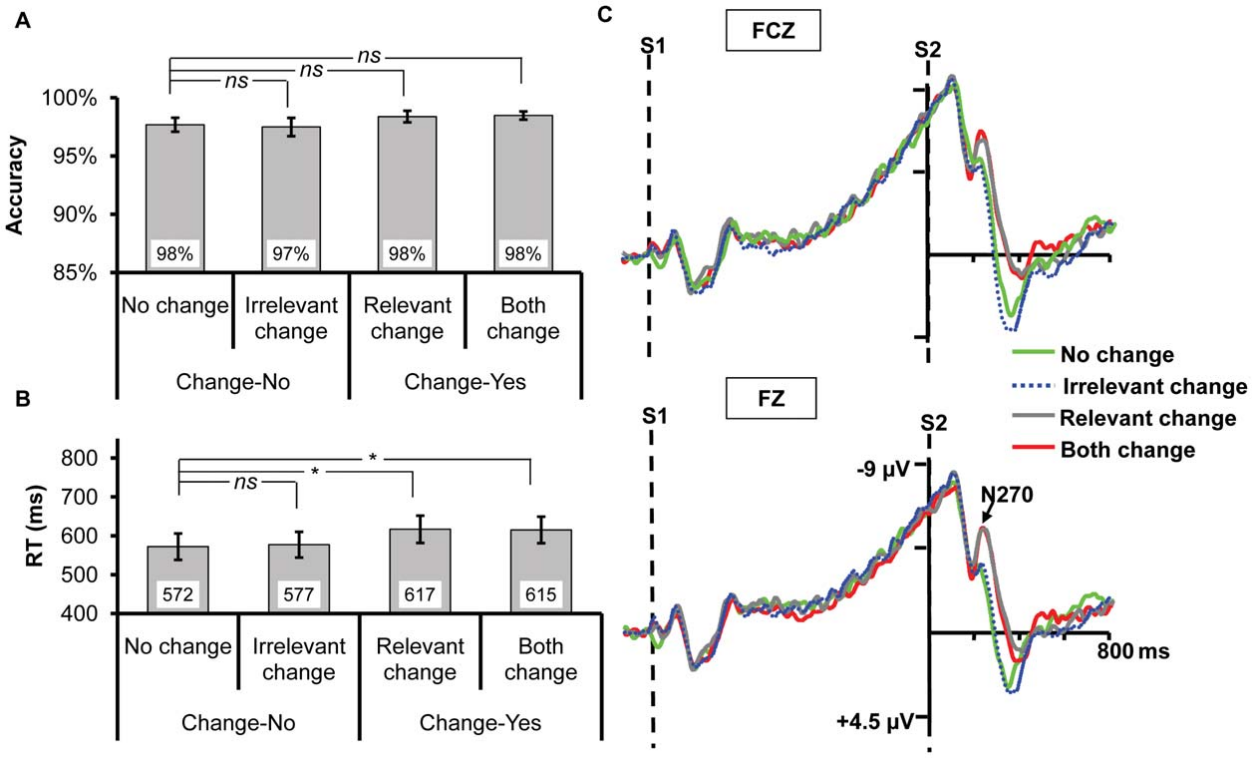
The results for Experiment 2a. (A) and (B) The accuracy and reaction times for the four types of change, respectively. The * and *ns* show the result of planned contrast between the three types of change and no change. * means the difference is significant, whereas *ns* means the difference is nonsignificant. (C) The ERPs recorded at FZ and FCZ. Only the relevant change and both change elicited N270 relative to no change, no difference existed between irrelevant change and no change.

A one-way ANOVA yielded a significant main effect of change type on RT (Figure 3B), *F*(3,39) = 15.765, *p*<0.001, while no effect on accuracy (Figure 3A), *F*(3,39) = 0.915, *p = *0.402. Planned contrast showed that there was no difference between irrelevant change and no change on accuracy or RT, *p*s >0.4; both the RTs of relevant change and both change were longer than that of no change, *F*(1,13) = 17.537, *p* = 0.001, *F*(1,13) = 24.983, *p*<0.001, respectively. The other ones were nonsignificant, all *p*s >0.2.

#### ERP Data

As shown in Figure 3C, only the change of target dimension elicited N270 while the waveform for the irrelevant change was almost identical to the no change. The two-way ANOVA revealed a significant main effect of change type, *F*(3,39) = 6.776, *p* = 0.011; the other ones were nonsignificant, all *p*s >0.3. Planned contrast showed that there was no difference between irrelevant change and no change, *F*(1,13) = 0.191, *p* = 0.670, suggesting that the irrelevant orientation change did not elicit N270. In contrast, similar to Experiment 1, both the amplitudes of relevant change and both change were higher than that of no change, *F*(1,13) = 6.531, *p* = 0.024, *F*(1,13) = 6.717, *p* = 0.022, respectively, indicating N270 was elicited when the target dimension changed.

Contrast to the automatic extraction of irrelevant color (high-discriminable information) demonstrated in Experiment 1, both behavioral and ERP results of the current experiment did not reveal any evidence supporting the automatic selection of the fine-grained gap orientation. Instead, it needs explicit task demand (e.g., to be the target dimension, as shown in Experiment 1).

## Experiment 2b: Short Maintenance Time between S1 and S2

Although the results of Experiment 1a (97%, gap-orientation) and 2a (98%, color) suggested that the fine-grained information as target feature did not decay quicker from VSTM than color, and the results Experiment 1b implied that it could not decay from iconic memory either, the absence of automatic extraction of the irrelevant fine-grained information in Experiment 2a may be due to the representation decay from VWM during the long blank interval between S1 and S2. This is because the maintenance of the fine-grained information is resource-demanding [10], [64]–[66], [ but see 7] and it receives less resource as a target-irrelevant dimension compared to the target dimension. To test this alternative, the current experiment limited the blank interval between S1 and S2 to 300 ms considering visual iconic memory lasts approximately 250 ms after the stimuli's offset [67]. If the fine-grained information can be automatically selected into VWM, it could not decay in such a short time period.

### Methods

Fourteen (8 females) students (Mean age 25.2 years) participated in the experiment. The blank interval between S1 and S2 was reduced to 300 ms. The participants provided written and informed consent before experiments, and all procedures were approved by the Research Ethics Board of Zhejiang University. The other ones were identical to Experiment 2a. An average of 10.6% of trials was excluded from further analysis. Two participants (1 male) were removed from the analysis for too many artifacts (over 30% of trials).

### Results and Discussion

#### Behavioral Data

**Figure 4:**
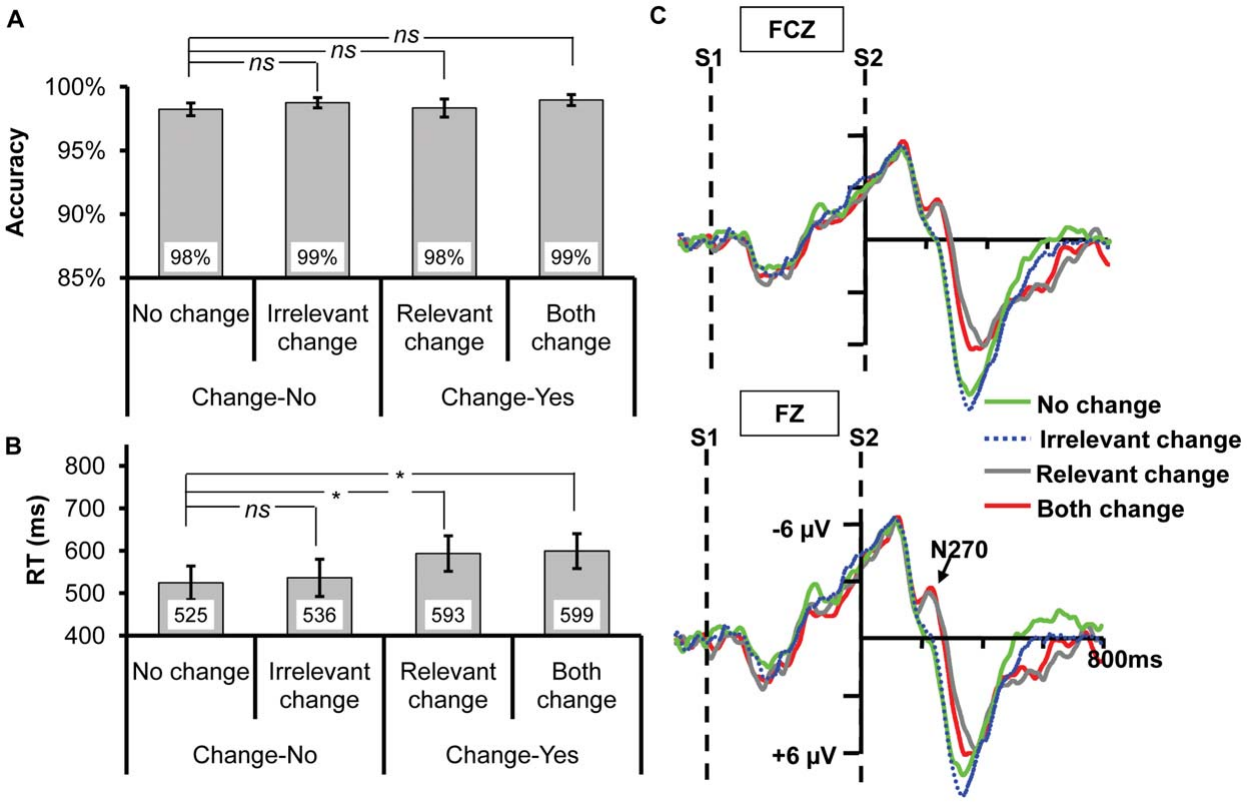
The results for Experiment 2b. (A) and (B) The accuracy and reaction times for the four types of change, respectively. The * and *ns* show the result of planned contrast between the three types of change and no change. * means the difference is significant, whereas *ns* means the difference is nonsignificant. (C) The ERPs recorded at FZ and FCZ. Only the relevant change and both change elicited N270 relative to no change, no difference existed between irrelevant change and no change.

As revealed in Experiment 2a, a one-way ANOVA yielded a significant main effect of change type on RT (Figure 4B), *F*(3,33) = 25.242, *p*<0.001, while no effect on accuracy (Figure 4A), *F*(3,33) = 0.480, *p = *0.670. Planned contrast showed that there was no difference between irrelevant change and no change on accuracy or RT, *p*s >0.13; both the RTs of relevant change and both change were longer than that of no change, *F*(1,11) = 42.566, *p*<0.001, *F*(1,11) = 36.454, *p*<0.001, respectively. The other ones were nonsignificant, all *p*s >0.23.

#### ERP Data

Consistent with Experiment 2a, Figure 4C showed that no N270 was evoked under the irrelevant change of orientation. The two-way ANOVA revealed a significant main effect of change type, *F*(3,33) = 14.784, *p*<0.001, while a non-significant main effect of electrodes, *F*(1,11) = 1.687, *p = *0.221; the interaction between the two was significant, *F*(3, 33) = 4.608, *p = *0.014. Planned contrast showed that there was no difference between irrelevant change and no change, *F*(1,11) = 0.061, *p* = 0.809, indicating that the irrelevant orientation change did not evoke N270. In contrast, both the amplitudes of relevant change and both change were higher than that of no change, *F*(1,11) = 32.585, *p*<0.001, *F*(1,11) = 11.196, *p* = 0.007, respectively, indicating that N270 was elicited when the target dimension changed.

Although using 300 ms iconic memory may dominate the current task (yet the elicitation of N270 by relevant change suggested VSTM was at least partially involved in the task), we considered that this setting provided us one condition maximizing the chance to observe the automatic extraction of fine-grained information. Therefore, the consistent behavioral and ERP results between Experiment 2a and 2b suggest that the non-automatic extraction of irrelevant orientation is not due to the information decay of the gap orientation from VWM. Besides,

## Experiment 2c: Equivalent Change-degree between Color and Orientation

The absence of automatic extraction in Experiment 2a and 2b may be alternatively related to the unbalanced change signal between the two types of information. That is, the change of gap orientation was rather weak relative to that of color. In Experiment 2c we equalized the perceptual change-degree between color and orientation to testify whether the similar result pattern can be obtained.

### Methods

Sixteen (7 females) students (Mean age 24.5 years) participated in the experiment. All participants provided written and informed consent before experiments, and the procedures were approved by the Research Ethics Board of Zhejiang University. Larger Landolt-Cs were used as stimuli (2.95°×2.95° of visual angle from a viewing distance of 70 cm). The landolt-C had two possible orientations: 0° and 180° (1.06° of visual angle for gap's height, yet pilot experiment revealed that it still needs focal attention to be processed). Our pilot experiment, in which the blank interval between S1 and S2 was shortened into 150 ms while color or orientation was displayed in separated blocks, showed that participants detected the change of color and orientation equally efficient in a perceptual detection task (accuracy: 0.95 vs. 0.96; *t*(11)<1; RT: 563 ms vs. 590 ms; *t*(11) = 1.2, *p* = 0.251). The other aspects were identical to Experiment 2a. An average of 15.7% of trials was excluded from further analysis. One participant (1 female) was removed from the analysis for too many artifacts (over 30% of trials).

### Results and Discussion

#### Behavioral Data

**Figure 5:**
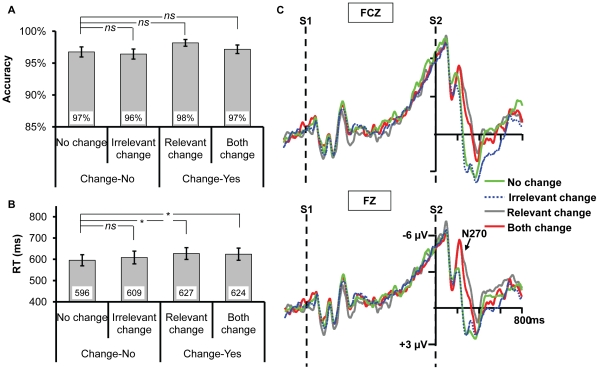
The results for Experiment 2c. (A) and (B) The accuracy and reaction times for the four types of change, respectively. The * and *ns* show the result of planned contrast between the three types of change and no change. * means the difference is significant, whereas *ns* means the difference is nonsignificant. (C) The ERPs recorded at FZ and FCZ. Only the relevant change and both change elicited N270 relative to no change, no difference existed between irrelevant change and no change.

A one-way ANOVA found a significant main effect on RT (Figure 5B), *F*(3,42) = 4.137, *p* = 0.036, while no difference was revealed on accuracy (Figure 5A), *F*<1. Planned contrast showed that there was no difference between irrelevant change and no change on accuracy or RT, *p*s >0.122; the RTs of relevant change and both change were slower than that of no change, *F*(1,14) = 12.364, *p* = 0.003, *F*(1,14) = 8.364, *p* = 0.012, respectively. The other ones were nonsignificant, all *p*s >0.13.

#### ERP Data

As evident in Figure 5C, no N270 was elicited under the irrelevant change condition. The two-way ANOVA revealed a significant main effect of change type, *F*(3,42) = 21.817, *p*<0.001. Planned contrast revealed no difference existed between irrelevant change and no change, *F*(1, 14) = 0.266, *p* = 0.614. Both the amplitudes of relevant change and both change were higher than that of no change, *F*(1,14) = 25.126, *p*<0.001, *F*(1,14) = 19.406, *p* = 0.001, respectively, indicating that N270 was evoked when the relevant dimension changed. The other ones were nonsignificant, all *p*s >0.330.

Consistent with the findings of the Experiment 2a and 2b, the current experiment still did not find any evidence on the automatic extraction of the irrelevant fine-grained information. To this point, the three experiments in Experiment 2 together suggest that the fine-grained information can not be extracted into VWM regardless of task demand but needs top-down control, supporting the analogy hypothesis.

## Experiment 3: Dissociated Extraction Was Not Restricted to a Specific Feature Dimension

Experiment 1 and Experiment 2 suggest there are dissociated mechanisms of information extraction for VWM. However, the distinctions in automatic extraction may merely reflect how a specific feature dimension is processed. To address the generability of our findings, in Experiment 3 different types of irrelevant change were produced by varying the same dimension of the basic feature. Previous research showed that the conjunction of homogenous basic features (e.g., color-color conjunction, two different colors with relative spatial relation between them) within an object can only be resolved by focal attentive processing [68]. We thus took the change of *color-color conjunction* within an object as the irrelevant detailed information change.

**Figure 6:**
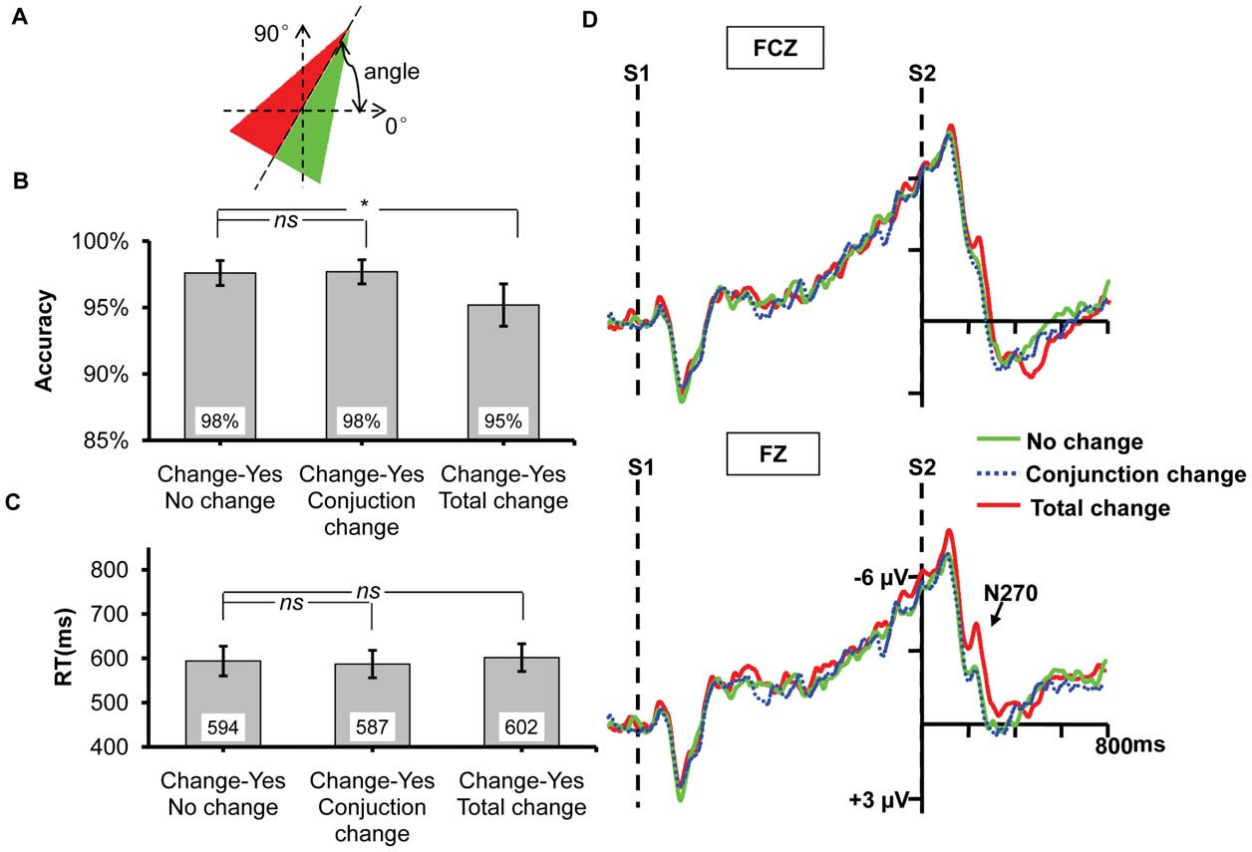
Example of the stimuli and the results for Experiment 3. (A) Orientated isosceles triangles used in the current experiment. (B) and (C) The accuracy and reaction times for the three irrelevant change conditions while triangle's orientation kept constant, respectively. The * and *ns* show the result of planned contrast between the three types of change and no change. * means the difference is significant, whereas *ns* means the difference is nonsignificant. (D) The ERPs recorded at FZ and FCZ. Total change elicited N270 relative to no change, whereas no difference existed between conjunction change and no change.

Each object contained two colors (see Figure 6A) which were irrelevant to the memory task, while the orientation of the triangle as the relevant dimension. For irrelevant information being resolved at the parallel stage of perception, the two colors within each object were replaced with new ones. For irrelevant information requiring focal attentive processing, the two colors contained in the object were held constant, but their relative positions within the object were swapped. If the automatic extraction in Experiment 1 is attributed to the fact that there is dedicated resource for retaining color information, then we should observe automatic extraction regardless of change type. However, if the information extraction in VWM is determined by the stage of perceptual processing at which the irrelevant information is selected, then the influence of the irrelevant change should only be observed for new colors, but not for new conjunction.

### Methods

#### Participants & Stimuli

Sixteen (7 females) students (Mean age 23.2 years) participated in the experiment. They all provided written and informed consent before experiments, and the experimental procedures were approved by the Research Ethics Board of Zhejiang University. Orientated isosceles triangles with an apex angle of 24.8° were used as stimuli. The length of midline of each triangle was 1° of visual angle. The orientation of the triangle was defined by the angle between the midline of the triangle and the horizontal axis (see Figure 6A). There were 12 different angles, starting from 0° and being selected every 30°. The triangle was divided into two parts along the midline, each part with a distinct color which was selected from a pool of seven colors. The colors in the current experiment were identical to those in Experiment 1 and 2 except that white was replaced by cyan (0, 180, 180).

#### Procedure & Design

Participants were instructed to remember the orientation of a triangle (S1), and then justify whether the orientation of S2 was the same to that of S1 while ignoring any color change. In 50% of the trials the orientation of triangle was changed. There were three types of irrelevant color-change: *no change*, colors of the triangle keeping constant; *conjunction change*, two colors contained in the object keeping constant, but their positions being swapped; *total change*, the two colors contained in the triangle being replaced by two new ones from the color pool.

A 2 (change of target feature: match, mismatch) ×3 (change of irrelevant feature: no change, conjunction change, and total change) within-subject design was adopted. Participants completed 80 trials under each of the 6 conditions, resulting in a total of 480 trials which were presented randomly. The experiment was divided into 6 blocks with 8-minute break in between, which lasted about 1 hour totally. Here we showed the data of the triangle's orientation being kept constant (i.e., relevant dimension did not change) since only this part was mainly related to our question (note clear N270 was evoked when triangle's orientation changed regardless of the irrelevant feature change). An average of 9.7% of trials was excluded from further analysis. Three participants (1 female) were removed from the analysis for too many artifacts (over 30% of trials).

The other aspects were identical to Experiment 1a and 2a.

### Results and Discussion

#### Behavioral Data

A one-way ANOVA led to a significant main effect of change type on accuracy (Figure 6B), *F*(2,24) = 4.286, *p* = 0.045; yet no effect on RT (Figure 6C), *F*(2,24) = 1.382, *p* = 0.271. Planned contrast showed that there was no difference between conjunction change and no change on accuracy or RT, *p*s >0.5; the accuracy of total change was significantly lower than that of no change, *F*(1,12) = 7.164, *p* = 0.02, suggesting that the total change distracted the judgment to the “no change” response; no difference was found between total change and no change on RT, *p*>0.5.

#### ERP Data

As evident in Figure 6D, the conjunction change did not evoke N270, while the total change elicited N270. This result profile was consistent with the behavioral results. The two-way ANOVA for the time window of 230–270 ms yielded a significant main effect of change type, *F*(2,24) = 6.766, *p = *0.005; both the main effect of electrodes and the interaction between the two were nonsignificant, all *p*s >0.2. Planned contrast showed that there was no difference between conjunction change and no change, *F*(1,12) = 0.191, *p* = 0.669; yet total change elicited a more negative component N270 than no change, *F*(1,12) = 8.364, *p* = 0.014.

Consistent with Experiment 1 and 2, both the behavioral and ERP results of Experiment 3 suggest that only the information processed at the parallel stage of perception can be automatically extracted into VWM, and this finding is not limited to the specific feature dimensions we tested in Experiment 1 and 2. Moreover, since the two types of irrelevant change were displayed randomly while focused on identical target feature, the current findings hence, to some extent, also suggested that the dissociated extraction was not due to different strategies that were used in the two types of information, for instance, the participants intentionally suppressed the change occurring in the fine-grained condition while not in the high-discriminable condition.

## Experiment 4: Dissociated Extraction in Multiple-item Condition

Experiment 4 had four aims. First, we tested the generality of our findings in a *multiple-item* condition. The findings revealed in the previous three experiments may only fit for the *one-item* condition since we usually remember multiple objects and most of the VWM studies thus used a multiple-item change detection task [1]–[13]. To this end, the current experiment required the participants to remember 3 colors considering VWM can hold 3∼4 of them [1], [2].

Second, we intended to verify the sensitivity of the task manipulations (the influence of the irrelevant change on performance) and indexes (behavior and N270) in measuring the information extraction of VWM, as so far the current experiment only adopted distinct colors as our tested high-discriminable information. Since irrelevant shape has been consistently shown to be automatically encoded into VWM [34], [35], [59], the current experiment thus adopted high-discriminable shapes as the task irrelevant dimension.

Third, we wanted to provide another piece of solid evidence that the absence of automatic extraction for the fine-grained information was not because the perceptual change-degree of it was too tiny relative to the high-discriminable information. In Experiment 4, while using the orientation of landolt-C in Experiment 1 and 2 as the irrelevant fine-grained information, *all* of the three orientations were changed when the irrelevant dimension changed. In contrast, only *one* of the three irrelevant shapes was changed in the high-discriminable condition. Our pilot experiment, in which the blank interval between S1 and S2 was shortened to 150 ms and either all the orientations from three displayed landolt-Cs or one shape from three presented shapes was changed randomly in the experiment, showed that the participants were equally efficient in detecting the perceptual change of landolt-C's orientation and simple shape (accuracy: 0.93 vs. 0.92; *t*(11)<1; RT: 690 ms vs. 715 ms; *t*(11) = 1.3, *p* = 0.21).

Fourth, we intended to further test the alternative that different processing strategies were adopted for the two types of information, considering the findings of Experiment 3 may be only restricted to the condition that color was the irrelevant dimension. Therefore, while we asked the participants to remember the same target feature color, the two types of irrelevant dimension (shape vs. orientation) were displayed randomly.

To reiterate, the analogy hypothesis predicted that only the irrelevant change of high-discriminable information (i.e., shape) could affect the behavioral performance and elicit N270.

### Methods

#### Participants & Stimuli

**Figure 7:**
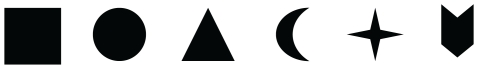
The six distinct shapes used in Experiment 4.

Sixteen (8 females) students (Mean age 23.5 years) participated in the experiment. All participants provided written and informed consent before experiments, and the procedures were approved by the Research Ethics Board of Zhejiang University. While the colored landolt-Cs in Experiment 1, 2a and 2b were employed for the fine-grained condition, a new group of colored shapes were used for the high-discriminable condition. The shape was selected from a set of 6 distinct shapes (Figure 7) while using the same set of colors in Experiment 1 and 2.

#### Procedure & Design

After a variable delay ranging from 1000 to 1400 ms, a fixation was displayed for 200 ms followed by 200∼300 ms blank interval. Then Stimulus Set 1 (S1) was displayed for 200 ms, followed by a 1000 ms blank period. Finally Stimulus Set 2 (S2) was always presented for 2000 ms regardless of whether the participant made a response or not, which was used to exclude any possible contamination of ERP response caused by the S2 offset. Both S1 and S2 contained 3 colored objects, which were displayed randomly in three of four possible locations with a distance of 1.8° to the center of screen. For these four locations, two of them were located horizontally while the other two vertically. In 50% of trials, S1 and S2 were colored shapes; while in the other 50% trials, they were colored landolt-Cs. Participants were instructed to remember the colors of the three objects while ignoring the irrelevant shapes or orientations, and justify whether the colors of S2 were identical to S1. Only the response accuracy was emphasized and recorded in the current experiment. This was because our pilot experiment found that it was difficult for the participants to respond as accurately and quickly as possible when S2 was always displayed for 2000 ms and our Experiment 1 to 3 showed that the influence of the irrelevant feature change was exhibited mainly on accuracy but not on RT.

The four change types were: S2 was either the same as S1 (*no change*), or different from S1 in one shape or three orientations (*irrelevant change*), or different from S1 in one color (*relevant change*), or S1 and S2 different in one color and one shape (belonging to the same object) or one color and three orientations (*both change*).

A 2 (irrelevant type: shape, orientation) ×4 (change type: no change, irrelevant change, relevant change, and both change) within-subject design was adopted. Participants completed 80 trials under each of the 8 conditions, resulting in a total of 640 trials which were presented randomly. The experiment was divided into 8 blocks with 5-minute break in between, which lasted about 1 hour totally. An average of 11.8% of trials for shape as irrelevant dimension and an average of 11.7% of trials for orientation as irrelevant dimension were excluded from further analysis. The other aspects were identical to Experiment 1.

### Results and Discussion

#### Behavioral Data

**Figure 8:**
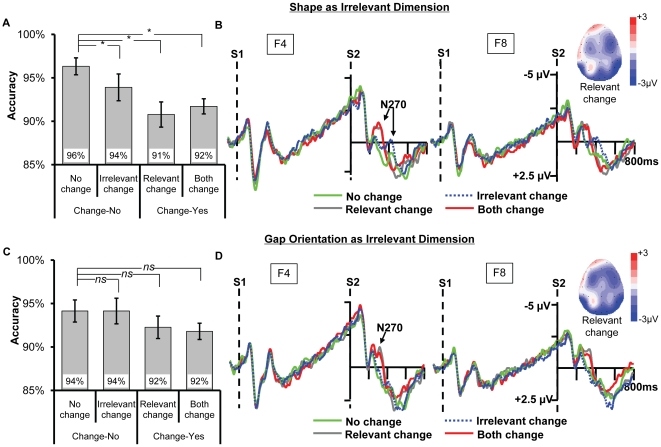
The results for Experiment 4. (A) and (C) The accuracy for the four types of change for shape as irrelevant feature and gap's orientation as irrelevant feature, respectively. The * and *ns* show the result of planned contrast between the three types of change and no change. * means the difference is significant, whereas *ns* means the difference is nonsignificant. (B) and (D) The ERPs recorded at F4 and F8, with the topography of relevant change (320 ms). Compared to no change, all the other three changes elicited N270 when shape was the irrelevant feature, yet only the relevant change and both change elicited N270 when orientation was the irrelevant feature.

The overall accuracy of the current study (0.93) is lower than that in Experiment 1 (0.97, *p*<0.005), 2 (0.98, *p*<0.001) and 3 (0.98, *p*<0.001), suggesting the current task is more demanding for VWM. A two-way ANOVA with irrelevant type and change type as factors (Figure 8A & 8C) showed that the main effect of irrelevant type was not significant, *F*(1,15)<1, suggesting that the performance of detecting color-change was equal in the two types of objects. The main effect of change type was significant, *F*(3,45) = 4.262, *p* = 0.031, and importantly, it was modulated by the irrelevant type by showing a significant interaction between the two factors, *F*(3,45) = 3.095, *p* = 0.036. To elaborate this interaction, we conducted separated planned contrast for the two types of irrelevant feature.

When shape was the irrelevant feature (Figure 8A), planned contrast showed that the accuracy of the irrelevant change was significantly lower than no change, *F*(1,15) = 7.097, *p* = 0.18. This result suggested that the irrelevant shape was encoded into VWM and impaired the detection of colors. Both the accuracy of relevant change and both change were also lower than no change, *F*(1,15) = 11.362, *p* = 0.04, *F*(1,15) = 32.819, *p*<0.01, respectively. However, when orientation was the irrelevant feature (Figure 8C), planned contrast showed that there was no difference between irrelevant change and no change, *F*(1,15) = 0. The other ones were also non-significant, *p*s >0.1.

#### ERP Data

Instead of analyzing FZ and FCZ in the previous three experiments, the current experiment analyzed the N270 in F4 and F8 because N270 was most obvious in these two sites across conditions (see the topography in Figure 8B and 8D). As shown in Figure 8B and 8D, the irrelevant change of shape elicited N270 although its latency was delayed relative to the relevant change and both change conditions, yet the irrelevant change of orientation did not evoke N270.

We conducted separate two-way ANOVA for the irrelevant shape and the irrelevant orientation trials. The latency of N270 (Mean peak latency 290 ms) was delayed in the relevant change relative to the one-item condition (Mean peak latency 230 ms), which may be because of the increasement of comparison difficulty. Therefore, a time window of 260–380 ms was used to measure the effect of change for the two types of irrelevant features except for the irrelevant shape change. As the N270 was further delayed in the irrelevant shape change (Mean peak latency 400 ms), which may suggest in a resource-demanding condition the comparison of the relevant feature is prior to the irrelevant one, a new time window of 330–450 ms was used to test its change effect.

When shape was the irrelevant feature, the two-way ANOVA by taking electrodes (F4, F8) and change type (no change, irrelevant change) as factors showed that the main effect of change type was significant, *F*(1,15) = 7.962, *p* = 0.013, suggesting that irrelevant change of shape elicited N270. Besides, the two-way ANOVA by taking electrodes (F4, F8) and change type (no change, relevant change, and both change) revealed a significant main effect of change type, *F*(2,30) = 4.486, *p* = 0.028. Planned contrast showed that both the relevant change and both change evoked N270, *F*(1,15) = 7.702, *p* = 0.014, *F*(1,15) = 4.790, *p* = 0.045. There was no difference between relevant change and both change, *F*<1. The other ones were non-significant, all *F*s <1.

When orientation of Landolt-C was the irrelevant feature, the two-way ANOVA by taking electrodes (F4, F8) and change type (no change, irrelevant change, relevant change, and both change) as factors showed that the main effect of change type was significant, *F*(3,45) = 5.369, *p* = 0.005. Planned contrast showed that there was no difference between irrelevant change and no change, *F*(1,15)<1, suggesting no N270 was elicited in the irrelevant change of orientation condition. In contrast, both the relevant change and both change evoked N270, *F*(1,15) = 7.374, *p* = 0.016, *F*(1,15) = 5.673, *p* = 0.031. There was no difference between relevant change and both change, *F*<1. The other ones were non-significant, all *p*s >0.05.

Although the multiple-item condition made the task more resource-demanding, both the behavioral and ERP results of Experiment 4 were consistent with the prediction of the analogy hypothesis, as well as the first three experiments using one-item condition. Therefore, the current experiment provides strong evidence that the dissociate extraction was not limited to the one-item condition, and the behavioral and N270 indexes can sensitively reflect the extraction mechanism. Moreover, our findings could not be explained by the unbalanced perceptual change-degree or different processing strategies.

## General Discussion

The goal of the current research was to investigate how the perceptual information is extracted into VWM. By manipulating the information type of the irrelevant dimension (high-discriminable information vs. fine-grained information) in a change detection task, we tested whether the object information was extracted via an integrated-object manner (object hypothesis), or a feature-based manner (feature hypothesis), or an analogous processing as that in visual perception (analogy hypothesis). We found that the VWM extraction mimics the way visual perception selects visual features from the outside scene. In particular, when the target relevant information changed, a more negative component N270 was elicited relative to no change regardless of information type while the response was delayed. However, only when the high-discriminable information which is automatically extracted at the parallel stage of perception was the irrelevant dimension, the irrelevant change affected the behavioral performance and elicited N270; the change of irrelevant fine-grained information did not entail any influence on the performance, or evoke N270. These results suggest that the high-discriminable information is automatically extracted into VWM regardless of task demand, whereas the fine-grained information needs explicit task instruction to be accessed. Together, these findings imply that dissociated extraction mechanisms exist in VWM for information processed via dissociated processes in perception (at least for the information tested in the current study), and thus support the analogy hypothesis.

### Dissociated Extraction Process in VWM

The current study for the first time provides clear-cut evidence demonstrating how the perceptual information is extracted into VWM. Previous research mainly explored how the information consolidation, maintenance, and comparison in VWM are executed [11]–[13], [69], [70], yet what happens at the first place before the perceptual information begins to be transferred into VWM remains largely unclear. Based on the findings from quantities of visual research which aimed at revealing the information selection mechanisms of visual perception, we adopted the high-discriminable information and fine-grained information, whose processing mechanisms in perception are largely accepted albeit there are still many enduring controversies, as the interested types of information to test the three hypotheses (i.e., object, feature, and analogy hypothesis) in the current study. Through six experiments, we showed that the automatic extraction of the high-discriminable dimension was not only restricted to basic shapes and orientations [12], [34], [35], [59], but also can be extended to distinctive colors; while the absence of the automatic extraction of the fine-detailed information was not limited to a specific dimension, two types of fine-grained information with totally different physical attributes exhibit the similar result patterns.

Therefore, the current research on the one hand provides strong converging evidence with previous behavioral, ERP and fMRI study [1], [12], [22], [31]–[36], supporting that the high-discriminable information is automatically extracted into VWM (Experiment 1, 3, and 4); on the other hand, it for the first time reveals that extracting the fine-grained information into VWM needs top-down control (Experiment 2a, 2b, 2c, 3 and 4). Therefore, both the object-based hypothesis and the feature-based hypothesis can only explain part of the above findings, with the former fitting for the high-discriminable information condition while the latter working well for the detailed information condition. In contrast, the analogy hypothesis, which claims that VWM and visual perception share similar processing mechanisms, predicts exactly the above results, suggesting that how the information is selected in visual perception may determine how it is extracted into VWM.

### Interaction between Visual Perception and VWM

The current finding consequently helps us understand the interaction between visual perception and VWM. Indeed, the current analogy hypothesis is put forward based on the shared neural mechanisms for information encoding and maintenance between visual perception and VWM. Here we further showed that at least for the two types of information tested in the current study, similar mechanisms exist for extracting information from a lower stage to a higher stage for VWM and visual perception. It is also worth noting that the dissociated extraction fashions revealed here bear an analogy with the reversed hierarchy theory (RHT) of visual perception [47]. Based on numerous previous visual studies, RHT claims that our vision begins with the automatic and parallel processing of basic features by spread attention; then the detailed information is updated into the already coded representation to form a coherent one via focal attention. Therefore, even though we only tested two types of visual information, it stands to reason to support the claiming that VWM is “a harnessing of processes that evolved for perceptual purposes” [71]. Furthermore, the current findings suggest that VWM actively engages in perceptual processing, dynamically receives and updates intermediated perceptual representations, with the extraction sequence depending on the selecting order of visual information at the perceptual stage.

### Object-based Selection of High-discriminable information in VWM

To investigate the extraction mechanism in VWM, the current research investigated the automatic selection of the irrelevant dimension, which, as a matter of fact, took the advantage of the object-based selection benefit. This benefit is a well shown phenomenon in visual perception ([72], [73]; see [74] for an excellent review), and it is also revealed in VWM by showing that at least for the objects formed by multiple simple features (e.g., simple color and orientation), the representations in VWM take the form of integrated objects [1], [12], [22], [31], [32], [75]. For instance, it has been found that remembering all the feature dimensions that an object contains is as accurate as only remembering a single feature dimension for multifeatured objects [1]. The current research pushes the above conclusion about the object-based selection to a further step, implying that this benefit may be only restricted to the high-discriminable information, which is the product of the parallel processing in visual perception. Though this implication is deduced from the influence of the irrelevant change on the behavioral performance as well as the ERP component, it also receives support from a newly proposed neural object-file theory of vision which is put forward based on a series of elegant and consistent fMRI studies on VWM [10], [76]–[79]. This theory claims that there are two dissociated processes in human visual system in sequence: object individuation and object identification. A fixed number of about four low resolution objects (or proto objects) are first selected and stored at the object individuation stage, and then the details of a subset of the already selected objects are encoded at the object identification stage. One key prediction of this theory is that the object-based selection happens at the individuation stage. Since the information processed at the parallel stage is in low resolution in essence [45], [47], therefore, only the high-discriminable information can be automatically extracted into VWM regardless of the task demand. Finally, a very recent study showed that information from subliminally presented simple shapes can be extracted into VWM and guides attention [80]. Since the shapes in that study were displayed for a very short time with both forward and backward masks, the information extracted into VWM is definitely low-resolution information which corresponds to the very fragile output of the parallel processing in visual perception. Therefore, it provides compelling evidence supporting the automatic extraction of the high-discriminable information in VWM.

To this point, the object-based selection benefit for the high-discriminable information appears to be fairly robust, since it receives both empirical and theoretical support using different approaches (behavioral, ERP, and fMRI), variable manipulations (e.g., irrelevant information change, the number of features to be remembered) and different memory load (one and three items in the current study and four item in Experiment 5 of [12]). In this case, it is a little bit surprising to see that two recent studies did not find any evidence for the automatic extraction of the irrelevant but high-discriminable information [37], [38]. In these studies, researchers looked into the consolidation and the maintenance phases of VWM, while they asked the participants not to remember the irrelevant dimension which also did not change through a trial. The null evidence on the extraction of the irrelevant dimension in the two studies may be due to the reason that the manipulations or the indexes were not sensitive enough to reveal the automatic extraction. Indeed, using a similar design as shown by the current study, a very recent work by the same group of researchers found that the change of the irrelevant high-discriminable information impacted the behavioral performance (see Experiment 5 in [12]), suggesting that it can be automatically extracted into VWM. Besides, the absence of the irrelevant dimension extraction in the fMRI study [37] is also possibly related to the stimuli they adopted. In particular, colored Gabor patches were used as materials in that study. However, the color and the Gabor patch possibly can not be well integrated since color is not the content of the Gabor patch itself but seems to be a covered-veil on it, or the two dimensions are overlapped without sharing the same outer contour (for a well demonstrated empirical evidence, see Experiment 2A in [81]). This possibility also receives support from a quite similar fMRI research using Gabor patches as materials, while the spatial frequency and the orientation of the patch itself as the dimensions of interest [36]. Not surprisingly, strong evidence supporting the extraction of the irrelevant dimension was demonstrated.

### The Absence of Automatic Extraction for Fine-grained Information & Relation to the Memory-perception Comparison

Realizing the fact that the null-influence of the irrelevant change of fine-grained information on performance always implies a possibility that the irrelevant fine-grained information can be encoded into VWM, the current study was very careful in providing converging evidence on the absence of the automatic extraction of the fine-grained information. For instance, we used physically different materials (orientation of Land-C and conjunction between colors), distinct measuring indexes (behavioral and ERP), and different memory load conditions (one and three objects) and experimental designs (block and random design) to test its automatic extraction. Consistently we did not find any evidence showing that this type of information can be automatically processed. Although to this point we believed we had provided sufficient and strong evidence on this aspect, one may still argue that the fine-grained information can be automatically extracted, but the current task setting was not appropriate to be used to explore the extraction mechanism of VWM. Instead, results from the current task setting reflected the memory-perception comparison mechanism of VWM. The null-influence of the performance and the N270-absense in the irrelevant change condition were because at the comparison stage the change signal of detailed information was suppressed yet the signal of high-discriminable information was not. While the current findings are indeed related to the memory-perception comparison process, we consider that the current and previous results, as well as the current task setting all suggest that the current study revealed the information extraction mechanism in VWM.

First, one of the most straight-forward ways to test the automatic extraction of the fine-grained information is examining the maintenance phase of VWM since the extracted information should be stored into VWM. Two recent ERP studies, which focused on the information maintenance phase of VWM [64], [65], actually tested this issue although the explored question was different from the current study. Most relevantly, by taking the colored landolt-C of Experiment 1, 2a and 2b as materials, we recently measured the information stored in VWM by adopting an ERP component contralateral delay activity (CDA) as an index [64], the amplitude of which reflects the information stored in VWM [82]. It has been demonstrated that human beings can remember 3∼4 distinct simple objects, which leads to higher CDA amplitude for 4 objects than 2 objects. However, we found that the storage of the Landolt-C's orientation led to no difference between 2 and 4 objects; whereas when the orientation was task irrelevant and the distinct color was the target (as shown in the current Experiment 2 and 4), we replicated the previous findings that 4 objects' amplitude was higher than 2 objects. These results strongly suggested that the irrelevant orientation of Landolt-C could not be stored in VWM automatically; otherwise, the no-difference result pattern between 2 and 4 objects should be observed even when color as the target feature. Moreover, the similar results were also replicated when colored random polygons, which contain fine-grained information on the shape dimension, were taken as materials.

Second, Hyun et al. [12] suggested that there are two processing stages for the VWM-perception comparison. The first stage is an unlimited-capacity comparison process. At this stage with the help of the top-down control, only the change of the task-relevant feature can pop out and capture attention. After the attention shifts to the position where the change happens, a limited-capacity process is executed. At this second stage, a deliberative comparison is conducted by comparing both the task relevant and irrelevant features of the object, and this process will be impaired if the irrelevant feature changes by, for instance, pressing the wrong response button. We consider that the current study is related to the second comparison stage, in which all the features contained in VWM are compared with the perceptual input. First, we revealed the performance-influence in the irrelevant change condition, which is a hallmark of the second stage. Second, the first comparison stage predicts that only the relevant change elicits significant N270, and the N270 latency is not affected by the number of the memorized objects (see N2pc results of [12] for example). Contrast to this prediction, our results showed that the irrelevant change evoked a N270 as clear and significant as the relevant change, and its peak latency was delayed in the multiple-item condition (Experiment 4) compared to the one-item condition (Experiment 1 to 3), suggesting N270 reflects the mechanism of second comparison stage. Therefore, the processing properties of the second comparison stage (deliberative comparison) allow us to explore the extraction mechanisms of VWM, which also ensures us to use the current task setting and indexes to explore the extraction mechanism of VWM.

Third, in Experiment 4 the change-degree of the fine-grained information (690 ms) was equal to or slightly larger (in terms of RT) than that of the high-discrimination information (715 ms). Displaying these two change conditions randomly, we found the similar results as shown in Experiment 1 to 3. It is unreasonable to assume that both types of information were encoded into VWM yet the participants adopted different comparison strategies for them (e.g., suppressing the change-signal of the fine-grained information but not for the high-discriminable information), especially the same target feature was memorized. This is because at this stage it is the change-degree of information playing a critical role not the information type. The most workable answer to the findings about the irrelevant fine-grained information is that the irrelevant detailed information is not extracted into VWM. Taken together, we consider the current study provides clearly empirical evidence on the information extraction of VWM.

### Summary

In sum, we explored the information extraction process from visual perception to VWM by adopting two types of visual information (high-discriminable information vs. fine-grained information). The results revealed that there were dissociated extraction mechanisms for the two types of information, which mimics the dissociation in the perceptual processing. As a case study on VWM extraction mechanisms, we only tested two types of typical information in our real-life, more work needs to be done to explore how VWM interacts with perception and extracts perceptual information into its limited storage space.
